# Radiation-Induced Pneumonitis Following Stereotactic Body Radiation Therapy for Left Upper Lobe Squamous Cell Carcinoma

**DOI:** 10.7759/cureus.115615

**Published:** 2026-09-01

**Authors:** Azouba Gulraiz, Aayush, Keval Thakkar, Maulinkumar N Patel, Mrithulla Dillibabu Sujatha, Aanshi Kothari, Kaushalendra Mani Tripathi, Chinyereugo L Nwokocha

**Affiliations:** 1 Medicine, California Institute of Behavioral Neurosciences and Psychology, Fairfield, USA; 2 Internal Medicine, National Capital Region Institute of Medical Sciences, Meerut, IND; 3 Internal and Hospital Medicine, Moffitt Cancer Center, Tampa, USA; 4 Division of Research &amp; Academic Affairs, Department of Internal Medicine, Larkin Community Hospital, South Miami, USA; 5 Internal Medicine, The University of Texas Health Science Center, Houston, USA; 6 Internal Medicine, Tbilisi State Medical University, Saburtalo, GEO; 7 Medicine, Smt. Nathiba Hargovandas Lakhmichand Municipal Medical College, Ahmedabad, IND; 8 Internal Medicine, White River Health Medical Center, Batesville, Arkansas, USA; 9 Department of Medicine, Imo State University Teaching Hospital, Owerri, NGA

**Keywords:** lung cancer, lung collapse, radiation pneumonitis, squamous cell carcinoma, stereotactic body radiation therapy

## Abstract

This report describes the case of a 67-year-old male with a history of treated prostate cancer who developed a left upper lobe (LUL) squamous cell carcinoma, which was treated with stereotactic body radiation therapy (SBRT). After SBRT, radiation pneumonitis together with tumor regression led to bronchial obstruction and post-obstructive volume loss that culminated in a rare complete left lung collapse, underscoring an uncommon but serious complication of SBRT. The patient presented with progressive shortness of breath; imaging demonstrated post-obstructive volume loss and heterogeneous FDG avidity consistent with radiation pneumonitis. Corticosteroids were initiated, and symptoms stabilized over the ensuing weeks. The case highlights the need for close surveillance for radiation-induced lung injury, especially in patients with chronic obstructive pulmonary disease and a substantial smoking history. Careful patient selection and structured post-treatment imaging remain essential when SBRT is used for non-small cell lung cancer. Future work should identify predictive biomarkers and mitigation strategies for high-risk patients.

## Introduction

Lung cancer is a leading cause of cancer-related mortality worldwide, with non-small cell lung cancer (NSCLC) comprising approximately 85% of cases [1]. Stereotactic body radiation therapy (SBRT) is a standard treatment for early-stage NSCLC in patients unsuitable for surgery, offering high-dose, precise radiation with excellent local control [2,3]. However, radiation-induced lung injuries, such as pneumonitis and lung collapse, are recognized complications [4,5]. This report describes the case of a patient with left upper lobe (LUL) squamous cell carcinoma who developed lung collapse and radiation pneumonitis post-SBRT, highlighting diagnostic and management challenges.

## Case presentation

A 67-year-old male with a history of prostate cancer treated with external beam radiation therapy presented for routine follow-up. A chest CT scan revealed a 2.5-cm LUL suprahilar mass during evaluation for previously noted pulmonary nodules. The patient, a former smoker with a 40 pack-year history, was asymptomatic at the time. Bronchoscopy confirmed an endobronchial LUL mass, which was diagnosed as squamous cell carcinoma (PD-L1 expression 0%). Mediastinal lymph node biopsy (station 7) was negative for malignancy. Due to significant comorbidities, including chronic obstructive pulmonary disease (COPD) and cardiovascular disease, the patient was deemed inoperable. No significant autoimmune issue was noted.

Approximately one month after presentation, the LUL mass was treated with SBRT, 45 Gy in five fractions (BED₁₀ = 85.5 Gy). This slightly conservative schedule was chosen to spare the proximal airway and residual lung in a patient with COPD and a heavy smoking history, a risk-adapted approach used for central or high-risk lesions when a BED₁₀ ≥ 100 Gy cannot be delivered safely. CT performed two months later showed reduction in mass size (Figure 1).

**Figure 1:**
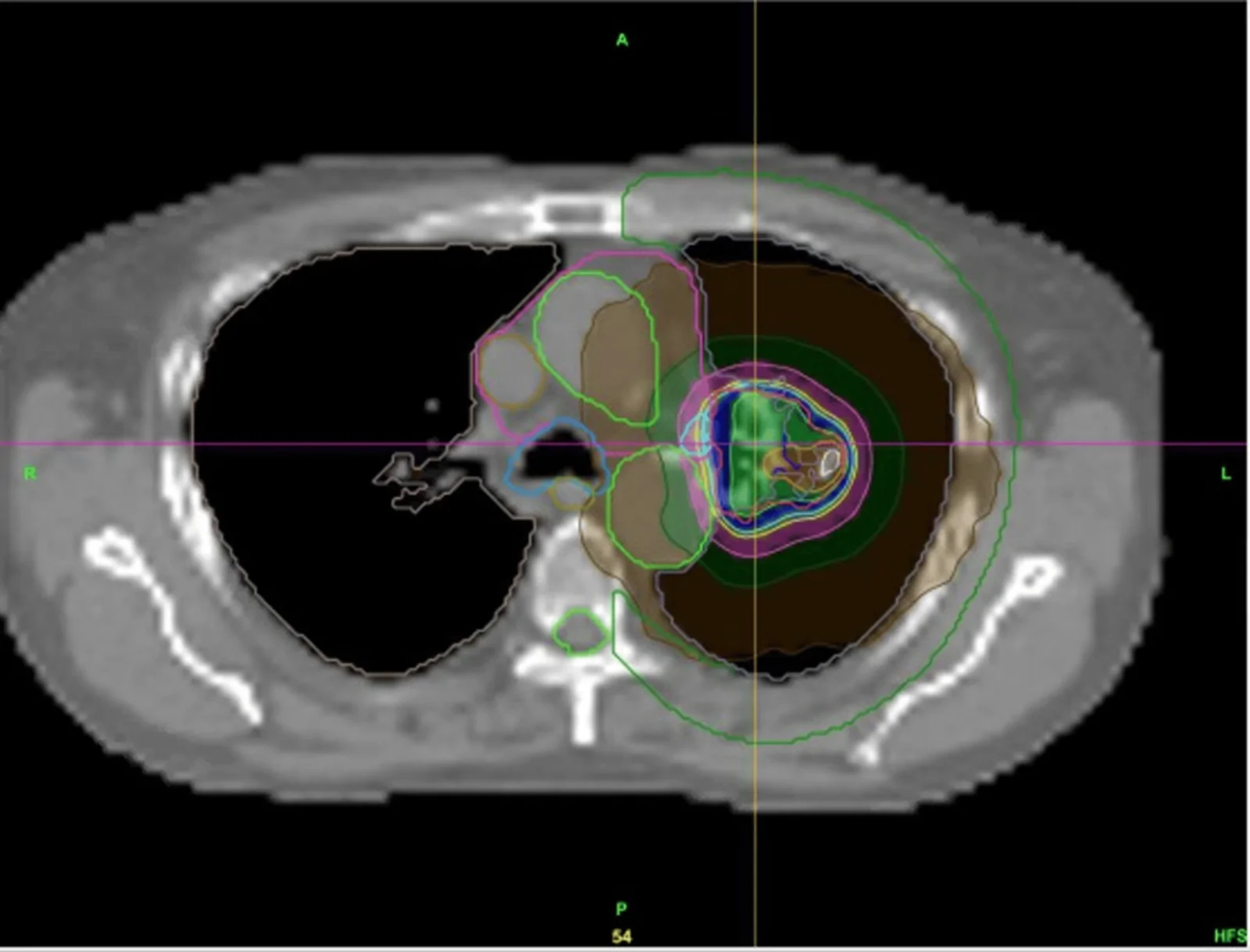
CT of the chest showing interval decrease in the left upper lobe mass following stereotactic body radiation therapy. Isodose color legend (color bar): Red: 100% prescription dose (45 Gy) Orange: 95% isodose line Yellow: 80% isodose line Green: 50% isodose line Blue: 20% isodose line Purple/magenta:10% isodose line

Approximately six months after SBRT, the patient reported a five-day history of shortness of breath (SOB), initially managed as a COPD exacerbation with prednisone (40 mg daily for five days), resulting in partial relief. One month later, the patient experienced a cerebrovascular accident (CVA) requiring left middle cerebral artery thrombectomy. A chest CT during hospitalization revealed an ill-defined heterogeneous mass-like lesion in the left suprahilar region with post-obstructive volume loss, suggestive of left lung collapse (Figure 2).

**Figure 2:**
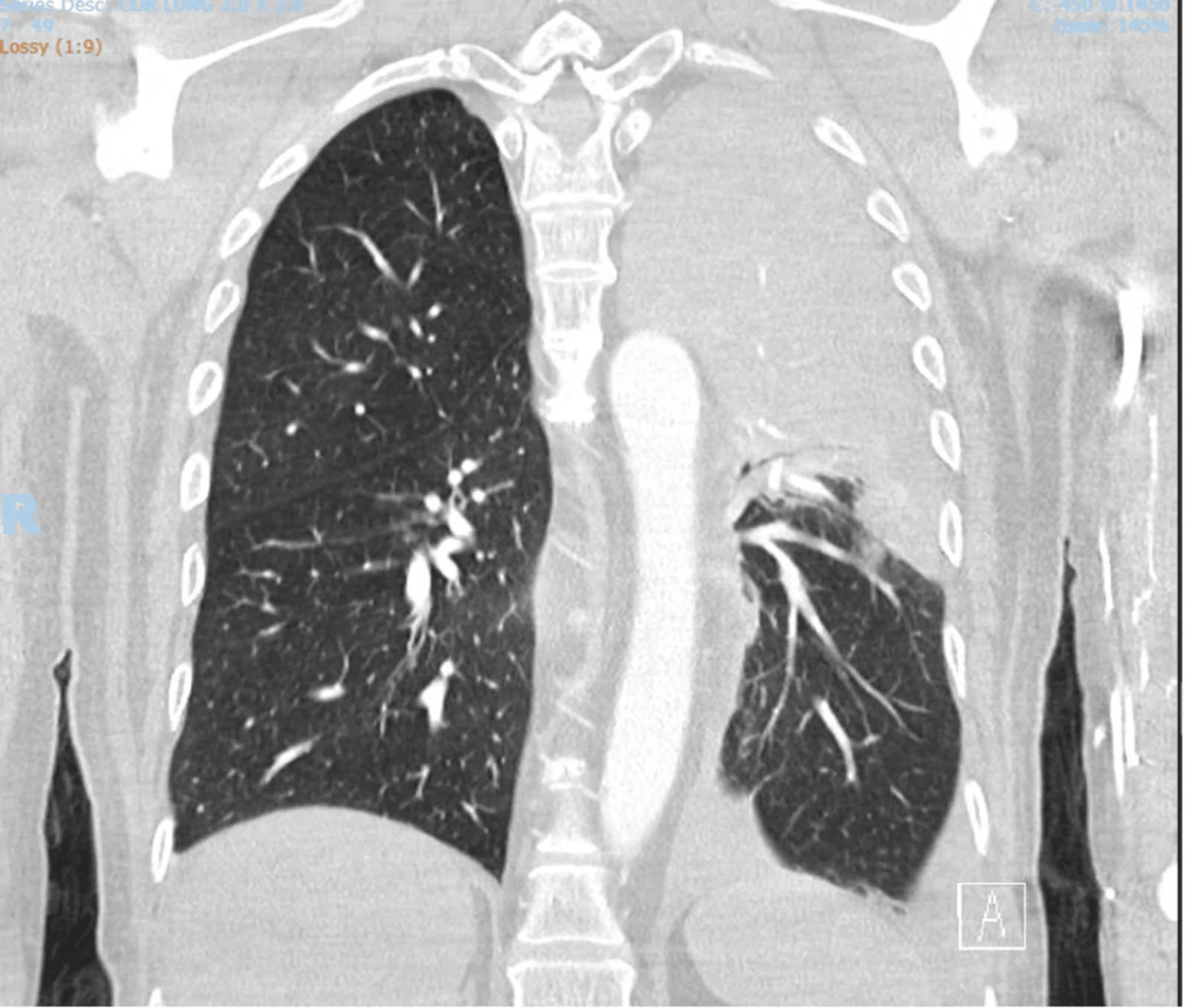
CT of the chest demonstrating left lung collapse with associated post-obstructive volume loss.

Post-SBRT imaging confirmed a reduction in tumor size, consistent with treatment response. Subsequent SOB and left lung collapse prompted further evaluation. CT showed post-obstructive volume loss, suggesting bronchial obstruction from tumor regression and/or radiation-induced fibrosis. Positron emission tomography/computed tomography (PET/CT) demonstrated heterogeneous FDG avidity in the treated field without a discrete focus of high uptake, a pattern supporting radiation pneumonitis rather than residual or recurrent malignancy. Repeat bronchoscopy found no viable tumor, further favoring a non-malignant cause for the collapse and pneumonitis.

The LUL squamous cell carcinoma was diagnosed using bronchoscopy with biopsy and staged as early-stage NSCLC. Approximately one month after presentation, the mass was treated with SBRT, 45 Gy in five fractions (BED₁₀ = 85.5 Gy), a risk-adapted schedule chosen to limit dose to the proximal airway and residual lung given COPD and a heavy smoking history.

Weeks later, the patient developed progressive SOB, first treated as a COPD exacerbation with corticosteroids, with only temporary improvement. Imaging then showed complete left lung collapse with post-obstructive volume loss. The collapse was attributed to radiation pneumonitis plus tumor regression producing bronchial obstruction; PET/CT heterogeneous avidity and a negative repeat bronchoscopy supported a non-malignant etiology. After a concurrent CVA, the patient required intubation and supportive mechanical ventilation. Radiation pneumonitis was treated with prednisone 1 mg/kg/day tapered over six weeks. No further radiation or systemic therapy was given. Symptoms stabilized over the following weeks; no additional oncologic treatment was required.

Over the subsequent months, the patient’s respiratory symptoms stabilized, with no further severe episodes of SOB. Follow-up imaging showed persistent post-treatment changes but no evidence of disease progression. Pulmonary function tests remained stable compared to baseline, indicating preserved lung function. The patient continued regular follow-up with oncology and pulmonology teams, with surveillance imaging planned every three to six months to monitor for recurrence or late radiation effects.

## Discussion

This case illustrates the complexities of managing radiation-induced complications in NSCLC patients treated with SBRT. Radiation pneumonitis, typically presenting 6-12 weeks post-treatment, is a well-documented toxicity, with risk factors including higher radiation doses, larger treatment volumes, and pre-existing lung disease [4-9]. Despite a maximum point dose of 46.93 Gy within acceptable limits, this patient developed pneumonitis, likely exacerbated by COPD and a 40 pack-year smoking history [6]. Studies indicate that radiation pneumonitis occurs in 10-30% of SBRT patients, with dose-volume analyses highlighting the role of lung dose in predicting toxicity [9]. Lung collapse, a less common complication, may result from bronchial obstruction due to tumor regression, fibrosis, or mucus plugging [5]. The temporal association with SBRT suggests a treatment-related etiology, though the precise mechanism remains unclear. Negative bronchoscopy and PET/CT findings ruled out tumor progression, aligning with studies comparing SBRT to surgical outcomes [10-14].

Management of radiation pneumonitis typically involves corticosteroids, with dosing tailored to symptom severity [8]. This patient’s response to prednisone underscores the importance of early recognition and treatment. The case highlights the need for multidisciplinary care involving oncology, pulmonology, and radiology to distinguish treatment effects from disease progression [10]. Advanced imaging, such as PET/CT, and repeat bronchoscopy were critical in confirming radiation-induced changes over malignancy recurrence. SBRT has been shown to achieve excellent local control for early-stage NSCLC, with clinical outcomes comparable to surgery in inoperable cases [2,3]. Comparative studies suggest that SBRT may be a viable alternative to lobectomy for stage I NSCLC, though complications such as pneumonitis require vigilant monitoring [13]. For operable patients, studies have explored the efficacy of SBRT relative to surgical resection [14]. In advanced NSCLC, high-dose radiotherapy regimens, such as those evaluated in RTOG 0617, highlight the balance between efficacy and toxicity [12]. The International Association for the Study of Lung Cancer (IASLC) staging guidelines further underscore the importance of accurate staging to guide treatment decisions [11]. Future research should explore predictive biomarkers for radiation toxicity and strategies to minimize complications in high-risk patients, such as those with pre-existing lung disease or prior radiation exposure [4-6,9].

## Conclusions

This case illustrates an uncommon but serious sequence after SBRT for a central, endobronchial LUL squamous cell carcinoma: radiation pneumonitis together with tumor regression produced bronchial obstruction and complete left lung collapse. The teaching point is anatomic rather than a generic “high-risk” label. Most SBRT candidates have COPD and a smoking history; what distinguished this course was a suprahilar endobronchial lesion in which treatment response itself could occlude the airway. Respiratory symptoms months after SBRT should prompt imaging that looks specifically for post-obstructive volume loss, not only parenchymal pneumonitis or recurrence. Steroids and supportive care coincided with symptom stabilization and no viable tumor on repeat bronchoscopy. As a single case, these observations cannot establish predictive factors or a preferred fractionation. However, they do support close clinical and radiographic follow-up after SBRT to central or endobronchial tumors, with a low threshold to evaluate new dyspnea for airway compromise.

## References

[1] Siegel RL, Miller KD, Jemal A (2020). Cancer statistics, 2020. CA Cancer J Clin.

[2] Timmerman R, Paulus R, Galvin J (2010). Stereotactic body radiation therapy for inoperable early stage lung cancer. JAMA.

[3] Nagata Y, Takayama K, Matsuo Y (2005). Clinical outcomes of a phase I/II study of 48 Gy of stereotactic body radiotherapy in 4 fractions for primary lung cancer using a stereotactic body frame. Int J Radiat Oncol Biol Phys.

[4] Yamashita H, Takahashi W, Haga A, Nakagawa K (2014). Radiation pneumonitis after stereotactic radiation therapy for lung cancer. World J Radiol.

[5] Rahi MS, Parekh J, Pednekar P (2021). Radiation-induced lung injury-current perspectives and management. Clin Pract.

[6] Shintani T, Kishi N, Matsuo Y (2021). Incidence and risk factors of symptomatic radiation pneumonitis in non-small-cell lung cancer patients treated with concurrent chemoradiotherapy and consolidation durvalumab. Clin Lung Cancer.

[7] de Groot PM, Chung JH, Ackman JB (2019). ACR Appropriateness Criteria(®) Noninvasive Clinical Staging of Primary Lung Cancer. J Am Coll Radiol.

[8] Hanania AN, Mainwaring W, Ghebre YT, Hanania NA, Ludwig M (2019). Radiation-induced lung injury: assessment and management. Chest.

[9] Barriger RB, Forquer JA, Brabham JG (2012). A dose-volume analysis of radiation pneumonitis in non-small cell lung cancer patients treated with stereotactic body radiation therapy. Int J Radiat Oncol Biol Phys.

[10] Debevec L, Debeljak A (2007). Multidisciplinary management of lung cancer. J Thorac Oncol.

[11] Goldstraw P, Chansky K, Crowley J (2016). The IASLC Lung Cancer Staging Project: proposals for revision of the TNM Stage Groupings in the Forthcoming (Eighth) Edition of the TNM Classification for Lung Cancer. J Thorac Oncol.

[12] Bradley JD, Paulus R, Komaki R (2015). Standard-dose versus high-dose conformal radiotherapy with concurrent and consolidation carboplatin plus paclitaxel with or without cetuximab for patients with stage IIIA or IIIB non-small-cell lung cancer (RTOG 0617): a randomised, two-by-two factorial phase 3 study. Lancet Oncol.

[13] Wang S, Wang X, Zhou Q (2018). Stereotactic ablative radiotherapy versus lobectomy for stage I non-small cell lung cancer: a systematic review. Thorac Cancer.

[14] Onishi H, Shirato H, Nagata Y (2011). Stereotactic body radiotherapy (SBRT) for operable stage I non-small-cell lung cancer: can SBRT be comparable to surgery?. Int J Radiat Oncol Biol Phys.

